# Quantitative Analysis of Radiation-Associated Parenchymal Lung Change

**DOI:** 10.3390/cancers14040946

**Published:** 2022-02-14

**Authors:** Edward Chandy, Adam Szmul, Alkisti Stavropoulou, Joseph Jacob, Catarina Veiga, David Landau, James Wilson, Sarah Gulliford, John D. Fenwick, Maria A. Hawkins, Crispin Hiley, Jamie R. McClelland

**Affiliations:** 1 Centre for Medical Image Computing, Department of Medical Physics and Biomedical Engineering, University College London, London WC1E 6BT, UK; a.szmul@ucl.ac.uk (A.S.); alkisti.stavropoulou.16@ucl.ac.uk (A.S.); j.jacob@ucl.ac.uk (J.J.); c.veiga@ucl.ac.uk (C.V.); j.mcclelland@ucl.ac.uk (J.R.M.); 2 UCL Cancer Institute, University College London, London WC1E 6BT, UK; dblandau@gmail.com (D.L.); Crispin.Hiley@crick.ac.uk (C.H.); 3 Sussex Cancer Centre, Royal Sussex County Hospital, Brighton BN2 5BE, UK; 4 UCL Respiratory Department, University College London Hospital, London NW1 2PG, UK; 5 Medical Physics and Biomedical Engineering, University College London, London WC1E 6BT, UK; james.wilson4@nhs.net (J.W.); s.gulliford@nhs.net (S.G.); m.hawkins@ucl.ac.uk (M.A.H.); 6 Institute of Systems, Molecular and Integrative Biology, University of Liverpool, Liverpool L69 3GE, UK; john.fenwick@liverpool.ac.uk

**Keywords:** radiotherapy-induced lung damage, lung cancer, deep learning

## Abstract

**Simple Summary:**

Radiotherapy is commonly used to treat inoperable locally advanced lung cancer. Despite the use of sophisticated modern planning and imaging techniques to target the tumour and minimise dose to normal lung tissue, patients can suffer from acute and chronic respiratory problems after treatment. Currently, our understanding of the impact that radiotherapy has on patients’ lungs is inadequate. We have, therefore, proposed a novel classification of the damage to the lung tissue, as seen on CT scans after a course of radiotherapy to a lung tumour. We have used deep learning algorithms to allow large numbers of CT scans to be labelled at the level of the individual voxel according to the degree of damage. The dose delivered to the tumour and the change in lung function of the patient after treatment both correlated well to the degree of radiological change measured. Our novel, automated classification combined with a dedicated image registration method has demonstrated an important clinical application that could be used to improve radiotherapy delivery in the future by allowing us to precisely track the changes seen after radiation treatment.

**Abstract:**

We present a novel classification system of the parenchymal features of radiation-induced lung damage (RILD). We developed a deep learning network to automate the delineation of five classes of parenchymal textures. We quantify the volumetric change in classes after radiotherapy in order to allow detailed, quantitative descriptions of the evolution of lung parenchyma up to 24 months after RT, and correlate these with radiotherapy dose and respiratory outcomes. Diagnostic CTs were available pre-RT, and at 3, 6, 12 and 24 months post-RT, for 46 subjects enrolled in a clinical trial of chemoradiotherapy for non-small cell lung cancer. All 230 CT scans were segmented using our network. The five parenchymal classes showed distinct temporal patterns. Moderate correlation was seen between change in tissue class volume and clinical and dosimetric parameters, e.g., the Pearson correlation coefficient was ≤0.49 between V30 and change in Class 2, and was 0.39 between change in Class 1 and decline in FVC. The effect of the local dose on tissue class revealed a strong dose-dependent relationship. Respiratory function measured by spirometry and MRC dyspnoea scores after radiotherapy correlated with the measured radiological RILD. We demonstrate the potential of using our approach to analyse and understand the morphological and functional evolution of RILD in greater detail than previously possible.

## 1. Introduction

Lung cancer is the leading cause of cancer-related death worldwide [1]. In inoperable locally advanced non-small cell lung cancer (NSCLC), chemo-radiation (CRT) is the standard treatment. Eligible patients are also offered up to 12 months of a Programmed death-ligand 1 inhibitor [2]. The lung is one of the most sensitive tissues to ionising radiation, and this limits the dose that can be delivered to lung tumours [3]. Radiation-induced lung damage (RILD) is divided into an acute, reversible phase, radiation pneumonitis, and a late, permanent, fibrotic phase [4].

Radiation damage is caused by both direct nuclear and mitochondrial DNA damage, and the generation of free radicals. Within minutes of irradiation, altered gene expression can be detected and growth factors, such as TGF-ß, PDGF and interleukin 1, are released [3]. Two distinct pathophysiological mechanisms of RILD have been described [4]. Classic RP occurs in-field and follows radiation to large volumes of lung parenchyma. A three-phase model of the histological changes (an early, intermediate and late phase) is described. The early or latent phase is visible only with electron microscopy, which reveals degenerative changes to type I and type II pneumocytes, thickened secretions of mucus from goblet cells, basement membrane swelling and changes to endothelial cells [4,5]. This early phase is dose dependent and does not generally occur at doses <10 Gy [5]. Damaged cells lead to cytokine release, e.g., TNF alpha, which leads to an acute inflammatory, exudative pneumonitis: the intermediate phase. The final, fibrotic phase is a result of pathological repair of the acute inflammatory insults. Fibroblasts produce collagen after stimulation by the acute phase proteins, particularly TGF-beta. This leads to a lack of lung elasticity, volume shrinkage and scarring. There is accompanying vascular damage, a decrease in type I pneumocytes and a return of type II pneumocytes.

This three-stage model, which presents a dose-dependent response to radiation, though widely cited, is a simplification. RILD is modulated by a host of genetic, environmental and psychological variables that are not yet well understood. An individual patient’s response to thoracic irradiation is somewhat unpredictable and sometimes out of proportion to the dose or volume of lung irradiated. Furthermore, radiological appearances and clinical symptoms are often poorly correlated [6]. It has been suggested that the three-stage model, derived largely from animal studies, or human studies where single dose, whole lung irradiation is administered, may not be clinically applicable to fractionated radiotherapy [5].

The incidence of symptomatic RILD is estimated to be 15–40% with a mortality rate of <2–4% [7,8]. Clinically, radiation pneumonitis manifests within weeks to months after the completion of radiotherapy. It presents with the classic triad of dyspnoea, non-productive cough and hypoxaemia. Pulmonary function tests usually reveal a restrictive defect due to volume loss. Gas exchange is also impaired and a fall in diffusion capacity may be seen.

Patient, tumour and treatment factors affect the likelihood of developing RILD [9] and its severity. Chemotherapy can have a synergistic effect with radiotherapy to exacerbate RILD [10]. Immuno-oncology (IO) therapy can cause pneumonitis, which is a widely recognised complication [11]. Rates of interstitial lung disease with IO agents are reported at rates between 2.7% and 3.5% [10]. It is likely that by priming the immune system, RT exacerbates this [12,13]. A secondary analysis of KEYNOTE-001 demonstrated that previous RT increased the incidence of pulmonary toxicity in patients receiving pembrolizumab [13].

A dose-response relationship can be plotted between Mean Lung Dose (MLD) and probability of radiation pneumonitis (or RP). Various Vx values (% of lung volume receiving ≥ X Gy) are associated with RP risk. There is no sharp dose threshold below which there is no risk of RP. Different dosimetric parameters are closely correlated within individual datasets, suggesting that there is no optimum threshold [14]. Widely accepted clinically-useful normal lung tissue tolerance doses allow the reasonably effective and safe deployment of radiation for lung tumours [14]. However, dose escalation remains an important ambition for radiation oncologists with the hope of improving survival [15]. The improved outcomes achieved for this patient cohort mean that clinicians must now pay more attention to the morbidity of treatment which patients can expect to endure for a greater period of time than their historical counterparts due to prolonged overall survival [16].

Despite a number of published classification systems [17,18,19,20,21], there are not clinically useful RILD classification to allow detailed, quantitative analysis of the radiological changes seen after RT, or that have known correlations with clinical or dosimetric measures. In order to address this, in 2018, our group published a novel method to quantify RILD with twelve automated image-based biomarkers to allow precise, objective and continuous measures of changes on CT scans following RT [22]. The biomarkers track changes to the size and shape of the lungs, the parenchyma and pleural thickness. The biomarkers have been used to track the evolution of RILD over time and were able to differentiate patients into two distinct subtypes according to the temporal pattern of radiological RILD manifested [23].

However, the biomarkers characterised parenchymal change by simply thresholding the lung tissue into ‘normal lung’ and ‘consolidation’ based on the HU value. This approach was an over-simplification and was unable to distinguish the different morphological subtypes of parenchymal change that could be seen in the images. Furthermore, the dosimetric correlations presented in [23] used only global DVH metrics and did not study the relationships between local dose and parenchymal change as, at that time, it was not possible to perform meaningful lung registrations due to the magnitude of the geometrical changes resulting from long-term RILD.

Our group has recently been undertaking work to address the issues raised above. Firstly, we developed a novel deformable registration method that enabled us to register the follow-up CT scans and the baseline scan despite the extreme geometrical and anatomical changes secondary to RILD occurring between the images [24]. This technique, rather than using the image intensities to guide the registrations, instead uses salient features between the images such as the lung boundary, the major airways and the blood vessels, to successfully register CT scans of the same patient up to 24 months apart. Secondly, we have developed a novel automated classification of parenchymal texture at the voxel-wise level, which enables us to study changes to parenchymal tissue due to RILD. To achieve this, we trained a deep-learning network using a two-stage approach akin to active learning. The technical details of this methodology are presented in an accompanying paper in this special issue [25].

The work presented in this paper utilises the recent advances outlined above and applied them to a clinical dataset to quantify parenchymal change due to RILD at a level of detail not previously performed. This includes longitudinal analysis of the proportion of the different tissue classes in the lungs, analysis of the relationships between the tissue classes at each time point and global dose metrics and measures of respiratory function, and investigating the relationship between local dose and changes to the parenchymal tissue over time.

## 2. Materials and Methods

IDEAL-CRT [26] was a multicentre, UK, phase 1/2, single arm, dose escalation trial in non-small cell lung cancer. Patients received isotoxically individualised tumour radiation doses of 63 to 71 Gy in 5 weeks or 63 to 73 Gy in 6 weeks, delivered concurrently with two cycles of cisplatin and vinorelbine. A total of 118 patients (Stage IIB-IV) were recruited from 9 UK centres. All patients gave informed, written consent. Scans of 46 patients who had CT scans at all follow-up points (3, 6, 12 and 24 months post RT) were available. For the baseline scans, a diagnostic CT obtained just prior to RT was used rather than the RT planning scan, as these were generally of superior quality. The RT planning scan, RT structures and dose distribution data were also available for all patients. MRC dyspnoea scores and spirometry data at baseline and follow-up were available for most patients (see Table 1 and Table 2 for full details).

**Figure 1:**
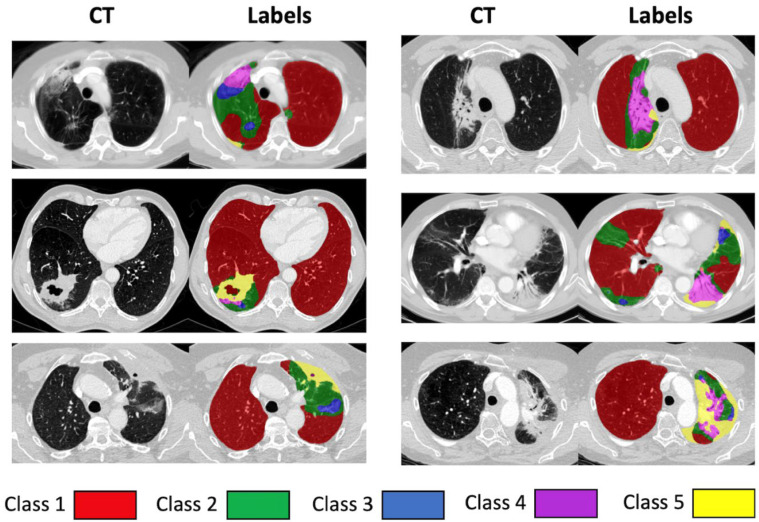
Examples of morphological classes used in parenchymal classification system. Left plain CT. Right automated labels. Class 1; Red, Class 2; Green, Class 3; Blue, Class 4; Magenta; Class 5; Yellow.

We have proposed a novel classification of lung parenchyma texture at the voxel-wise level and developed a deep learning-based method of its automated annotation. Following a detailed study of the texture changes seen in our patient cohort, and a review of established classifications in the literature [17,18,20,21,27,28,29,30,31,32], we devised a five-class classification based on the morphology and texture of the lung parenchyma. The full details are presented in our accompanying paper in this Special Issue [25]. Table 3 lists the different classes and Figure 1 shows examples of the classes on several CT slices.

To automate the classification, the data were divided into a development set (40 patients) and a hold-out test set (6 patients). An initial manual labelling of the development CT scans was performed by a radiation oncologist (E.C.) with review and refinement by a thoracic radiologist (J.J.). As the manual labelling was challenging, and involved some uncertainty in the labelling process, we decided to adopt a two-stage learning approach, akin to active learning. We used the initial manual labels on the development dataset to train an initial ensemble of Convolutional Neural Networks (CNNs), of which the results were combined to form an initial automatic labelling of the development data. The initial manual and automatic labels were carefully reviewed and edited to produce a single revised set of ground-truth labels for the second stage of training and evaluation. An expanded ensemble of CNNs were then trained on the revised labels, and the results were evaluated on the hold-out test set. The ground truth labels on the hold-out test set were generated entirely manually, but only after the revised ground truth labels on the development set had been generated to ensure they were consistent with the revised labels.

**Figure 2:**
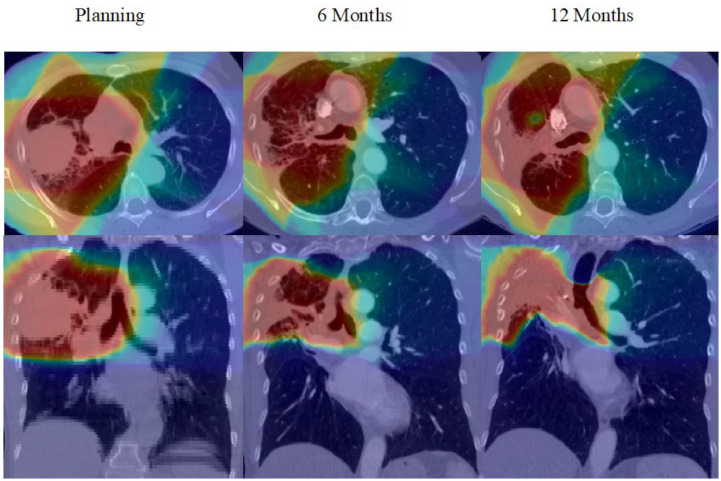
An example of how the dose was propagated from the planning scan onto the 6- and 12-month time points.

In order to propagate the Planning Tumour Volume (PTV) and local dose distribution onto the baseline and follow-up CT scans, the planning RT scans were registered with each of the five available diagnostic CT scans obtained before and following treatment; see Figure 2 for an example. This was performed using our recently developed novel deformable registration method, which aligns images based on the salient features between the images. Briefly, this method does not use the intensity information in the images to align the images, but instead uses segmentations of the lungs and major airways, together with blood vessels detected using a ‘vesselness’ filter [33]. These features are then used to drive a multi-channel deformable registration. The lung segmentations were performed manually and the airways segmentations were performed automatically using the open-source Pulmonary Toolkit [34] and subsequently reviewed and manually edited if needed using ITK-SNAP [35]. The ‘vesselness’ [33] of each scan was calculated using the Pulmonary Toolkit. The multi-channel registrations were performed using the opensource NiftyReg software [36]. For each patient, the planning scans were registered to all of the diagnostic CT scans, and the registration results were used to propagate the PTV and local dose distribution onto the scans at all time points. All registrations were manually reviewed and judged to be of sufficient quality, except for one patient, who could not be registered due to the severity of lung damage, meaning airways and vessels could not be aligned. Full details have been published elsewhere [24].

To analyse the prevalence of each parenchymal class at the different timepoints, the relative volume (as a percentage of the total lung volume) of each parenchymal class at every time point was calculated for each individual, and the mean values over all 46 patients were calculated and visualised as pie charts.

To analyse the evolution of the parenchymal classes over time, the difference in the relative volumes of the parenchymal classes was calculated between each follow-up CT scan and the pre-RT scan, for each individual. For this analysis, the voxels within the PTV were excluded from all timepoints so that presence of the tumour in the baseline scan did not influence the analysis and only the effects on lung tissue were studied. The registration results are required to transform the PTV on to each of the follow-up CT scans, so the one patient for whom the registrations failed was excluded from this (and all further) analysis. The differences in the relative volumes of the parenchymal classes, and their distribution over all individuals, were visualised using boxplots. A Friedman test was used to test for statistical differences between the distributions at different time points.

The relationship between the global dose and the change in the prevalence of the different tissue classes at each timepoints was analysed. For each follow-up CT scan, the Pearson correlation was calculated between the relative volume change of each tissue class and the following dosimetric parameters: V5, V10, …, V60, Mean Lung Dose (MLD), Max Lung Dose, the GTV volume and the PTV volume.

The relationships between the changes in the tissue classes and the changes in lung function were also investigated. Four different respiratory parameters related to lung function, FVC, FEV1, TLCO and MRC score, were measured at most timepoints for most patients (Table 1b). The changes in the respiratory parameters between each follow-up timepoint and the baseline measurements were calculated, and the Pearson correlation was calculated between the changes in respiratory parameters and the changes in tissue classes.

The relationship between the local dose distribution and the tissue classes distribution, and how this changed over the timepoints, was also explored. Each diagnostic CT scan was divided into subvolumes based on the propagated dose distributions, with a subvolume for each 5 Gy physical dose band up to 65 Gy, and then a final subvolume for all lung voxels receiving more than 65 Gy. For each patient and timepoint, the relative volumes of the tissue classes within each subvolume were calculated, and the average values were calculated for all patients. These results were visualised and qualitatively analysed using a stacked bar graph.

All statistical analysis was performed using Microsoft Excel for Mac Version 16.16.27, RStudio Version 1.2.1335 and IBM SPSS Statistics Version 27.

## 3. Results

The characteristics of the 46 patients included in this study are summarised in Table 1. The parenchymal tissue classes developed in our accompanying paper [25] are described in Table 3 and examples are given in Figure 1. The classes (1–5) were designed to represent texture with increasing density.

**Figure 3:**
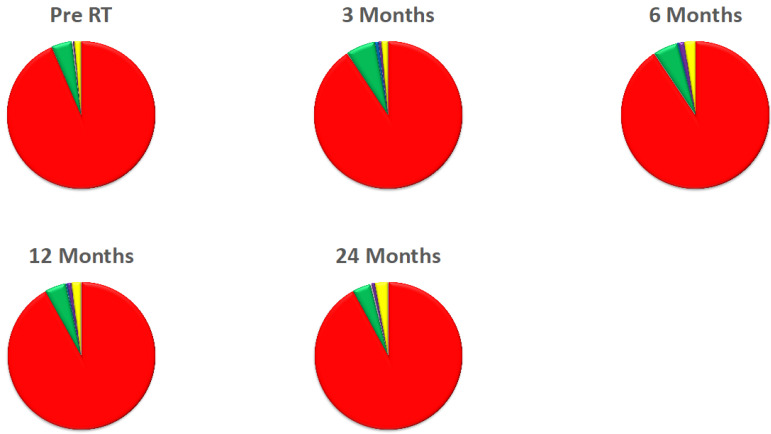
Mean % Volume of Tissue Classes for all patients in the entire lung volume including PTV, at each time point. Red = Class 1, Green = Class 2, Blue = Class 3, Purple = Class 4, Yellow = Class 5.

Figure 3 shows the mean proportion of each parenchymal class over all 46 patients at each timepoint. It can be seen that the prevalence of the different classes is broadly similar between the different timepoints, although the exact proportion of each class does vary between the timepoints. Class 1 is most prevalent for all timepoints and ranges from 90.6% at 6 months to 93.5% at the pre-RT scan. The next most prevalent class is Class 2, which ranges from 4.0–6.5%, followed by Class 5, which ranges from 1.4% to 2.9%. Note, as the PTV voxels were not excluded from this analysis, most of the Class 5 tissue in the pre-RT scan corresponds to the tumour. Classes 3 and 4 both represent very small proportions of the lungs, ranging from 0.25–0.74% and 0.29–1.25%, respectively.

**Figure 4:**
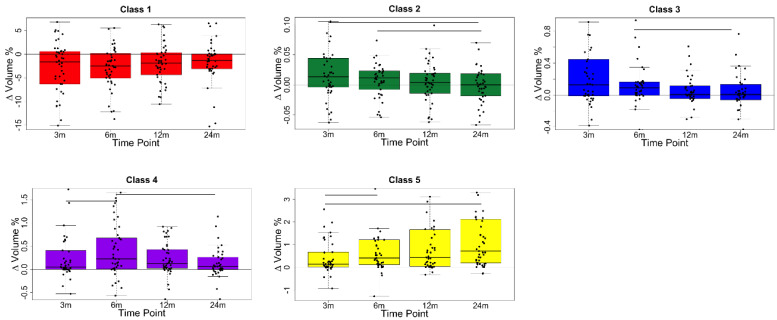
Boxplot showing difference in volumes of each class at follow up and baseline. Solid lines signify statistically significant (*p* < 0.05) differences between time points. The area within the PTV at each time point is excluded.

Figure 4 shows a boxplot for each parenchymal class, presenting the difference in the relative volume of that class between each of the follow-up CT scans and the pre-RT scan for the 45 patients that were successfully registered. Each of the classes displays its own distinctive temporal pattern. Prevalence of Class 1, which best represents undamaged tissue, shows a consistent decrease compared to pre-RT. This is most pronounced at 6 months and then gradually returns towards baseline at later timepoints. Classes 2 and 3, which represent ground glass and are, therefore, radiological markers of pneumonitis, are most prevalent at 3–6 months and then return towards baseline values at 24 months. Classes 4 and 5, which represent more solid textured parenchyma, behave differently from each other. Class 4 peaks at 6 months, while Class 5 continues to increase in prevalence up to 24 months. The latter correlates with the increased incidence of late lung fibrosis and collapse. The former may be an intermediary stage between the more acute Classes (2 and 3) and Class 5, representing evolving fibrosis. Friedman tests showed that there were statistically significant differences between the volumes at the time points connected by a horizontal line.

**Figure 5:**
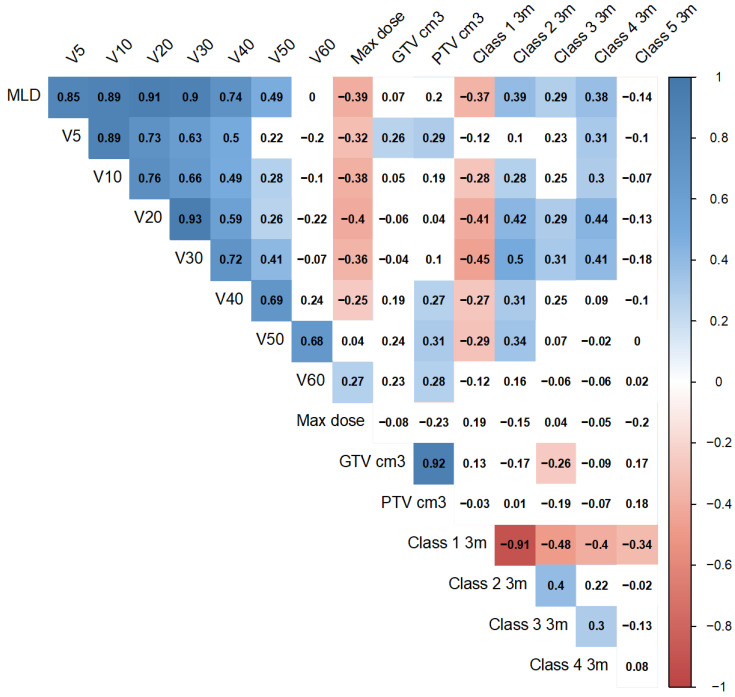
Pearson correlation matrix of global dose metrics and change in relative volume between pre-RT and 3-months post RT for each tissue class. VX = Lung VX Gray. MLD = Mean Lung Dose. PTV = Planning Target Volume, GTV = Gross Tumour Volume. Max dose = maximum prescribed dose (Gy). White boxes, *p* ≥ 0.1.

Figure 5 shows a Pearson correlation matrix for a number of global dose metrics and the relative volume changes of the different tissue classes at 3 months. Mild to moderate correlations are seen (up to r = 0.5). As expected, volume of Class 1 is negatively correlated with dose, while the other tissue classes show a positive correlation. The strongest correlation between the tissue classes is a negative correlation (r = −0.91) between the volumes of Class 1 and Class 2, suggesting that the decrease in default lung tissue at 3 months is mostly a result of an increase in ground glass texture (a classical radiological hallmark of radiation pneumonitis). The dosimetric variables that most strongly correlated with change in tissue class volume are lung V20–V50 Gy and mean lung dose. Lung V30 Gy was correlated with change in Classes 1, 2, 3 and 4 with r values of −0.45, 0.5, 0.31 and 0.42, respectively. Change in Class 5 shows the least strong correlations with global dose. Similar patterns are seen at other follow-up time points (see Figures S1–S3).

**Figure 6:**
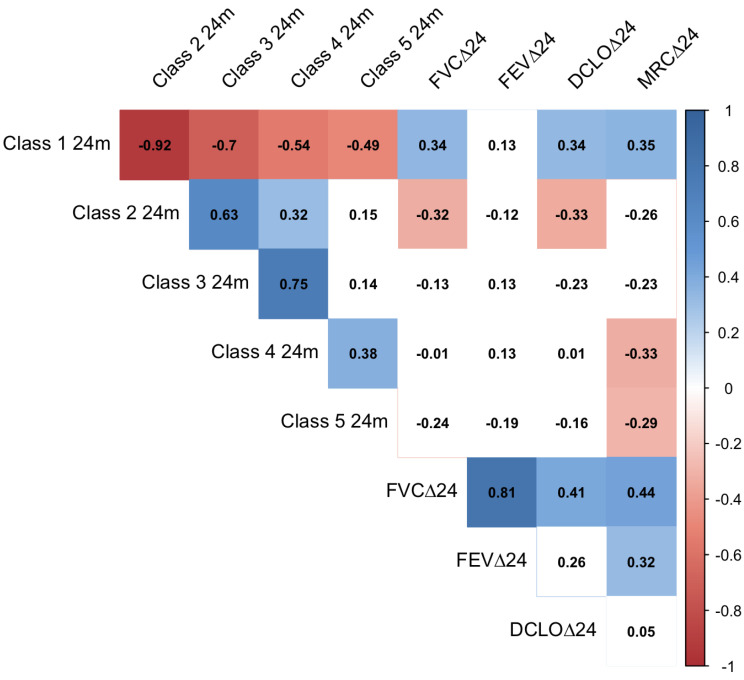
Pearson correlation matrix of respiratory metrics and change in relative volume between pre-RT and 24-months post RT for each tissue class. FVC = Forced Vital Capacity, FEV = Forced Expiratory Volume, DLCO = diffusing capacity for carbon monoxide, MRC = MRC dyspnoea score. White boxes, *p* ≥ 0.1.

Figure 6 shows a Pearson correlation matrix presenting the relationship between change in respiratory outcome metrics between baseline and 24 months and the changes in volume of tissue classes at 24 months. A moderate correlation was seen between change in Classes 1 and 2, and changes in the FVC, DCLO and MRC dyspnoea score. A weak correlation was observed between changes in volume of Classes 4 and 5 and changes in the MRC dyspnoea score. However, at earlier time points, the correlations were weaker. See Figures S4–S6 for the other time points.

**Figure 7:**
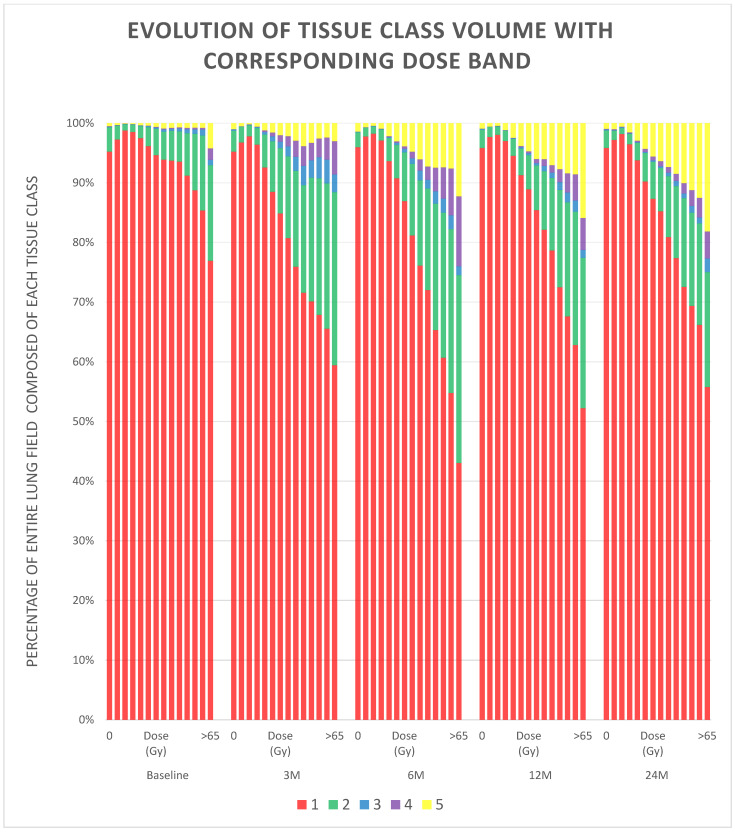
Stacked bar graph visualising the relationship between the local dose and the distribution of the tissue classes, and how this changed over the different timepoints. The different colours correspond to the different tissue classes and each column represents a 5 Gy dose bin from 0–5 Gy–60–65 Gy (with a final column for >65 Gy) from each of the different time points.

Figure 7 represents the relationship between the local dose and the distribution of the tissue classes, and how this changed over the different timepoints. It can be seen that the prevalence of tissue Classes 2–5 increases more in regions of higher dose, and remains fairly constant in regions of low dose, indicating that the changes to the tissue classes are indeed caused by radiation. It can also be seen that the dose response and temporal evolution is different for the different tissue classes. There is an initial increase in Class 2 at 3 months, which is approximately linearly related to local dose, and then a gradual decrease over later timepoints. There is also an increase in Class 3 at 3 months, but this occurs more evenly over the mid- and high-dose regions, and then rapidly decreases at later time points. Likewise, Class 4 increases at 3 months in the mid- and high-dose regions, but interestingly, decreases in the mid-dose regions but continues to increase in the high-dose regions at 6 months, and then gradually decreases in all regions at 12 and 24 months. Class 5 increases over all time points, but at 3 months, the increase is approximately the same in the mid- and high-dose regions, whereas at later time points, there is an increasingly linear relationship between the increase in Class 5 and the local dose.

## 4. Discussion

The relationships between radiotherapy dose, radiological changes and clinical outcomes are complicated. There are a large number of important clinical variables that make its analysis complex. Nonetheless, in this work, we have demonstrated that our novel classification system of lung parenchyma is a useful tool for studying RILD and uncovering patterns in its evolution and relationship to dose. Our classification system tracks longitudinal change that are related to both global and local prescribed RT dose. The global metrics with the strongest correlations were seen at 3 months and include r values of up to 0.5 between Class 2 and lung V30 Gy. Classes 1–4 all showed consistent mild to moderate correlation with a number of global dose metrics. Clinical outcomes (spirometry and MRC dyspnoea score) were available for most patients but only showed weak correlations with parenchymal changes. This is likely due to the number of confounders that can affect these measures. Spirometry is a summary measure of lung physiology and is determined by co-morbidities and patient compliance, amongst other things. Signals from spirometry that are directly affected by RILD can easily be lost amongst the noise of other variables [37]. The MRC dyspnoea score has even greater problems due to its subjective nature, and is significantly influenced by cardiac, orthopaedic and psychological morbidity as well as RILD and other respiratory conditions.

Given the complexity discussed above, this work was motivated by the recognition that the relationship between radiotherapy dose, radiological changes and clinical outcome is not yet adequately understood. There are several widely used clinical scoring systems for RILD based on a combination of symptoms and radiological severity [38]. The morphological changes are described imprecisely and terms such as ‘patchy’ and ‘dense’ are not formally defined. The clinical outcomes are categorised largely on their required therapeutic interventions and there is little emphasis on the functional impact on the patient [6].

Faria et al. have highlighted that two of the most commonly used scoring systems (RTOG/EORTC and NCI-CTC) are very poorly correlated with each other, and that radiological toxicity was rarely associated with symptoms [6]. Tucker et al. [38] have similarly shown that using NCI-CTC, CTCAE and RTOG as scoring systems for RILD give rise to markedly different normal tissue complication probabilities for a particular MLD, suggesting that the existing radiobiological models of RILD are not reliable.

A number of attempts have been made to improve upon these manual classification systems. Computational approaches have the advantages of allowing quantitative, continuous, objective and automated classification of radiological or functional lung damage. One of the most common approaches has been to use Hounsfield Unit (HU) density [31,32,39,40,41,42,43,44,45,46,47,48,49,50,51,52,53,54,55]. Palma et al. [52] performed deformable image registration on the phase of the planning scan with the lung volume most similar to the 3-month follow-up CT images of patients treated with SABR. Rigid registration was applied manually and then a modified B-spline Free Form Deformation algorithm was used to warp structures to achieve the required 3D displacement. Voxel HU density histograms were created and mean lung densities were derived. There was very poor correlation between HU density changes in the whole lung and the severity of physician graded radiological pneumonitis; however, local density changes around the PTV correlated strongly with increased radiological pneumonitis (Spearman’s r = 0.75).

A more recent example of the same technique [54] studied 31 patients receiving SABR to investigate the relationship between normal lung CT density changes with dose accuracy and outcome. Each patient was assigned a CTCAE RP grade. HU changes in 5 Gy dose bins from 5–45 Gy were assessed in the peri-tumoural region (ITV+ 3 cm margin). The 0–5 Gy lung volume was used as a baseline correction of the density changes. The average lung density changes in the peritumoural region for each of the 5–45 Gy dose bins were tabulated and compared across different dose algorithms. There was a strong positive relationship between peritumoural lung density changes and RP grade (Spearman’s r = 0.76). Positive correlation was also observed between RP and HU changes in the region covered by V20 for all algorithms (Spearman’s r ≥ 0.738). Additionally, V20, MLD and gEUD (generalised equivalent uniform dose) were significantly correlated with RP grade (*p* < 0.01).

Bernchou [32,56,57] et al. used a similar approach to investigate longitudinal change of HU density after IMRT for NSCLC. They found that normal tissue showed a significant increase in HU density after RT within even low dose areas. They noted that the evolution of changes differed between the low- and high-dose regions. Lung parenchyma receiving doses <45 Gy underwent a decrease in HU density after 3 months, while areas receiving 50–60 Gy became denser between 3–9 months before then decreasing again. Beyond 12 months, the density changes stabilise across all dose intervals. The bimodal time distribution supports a model of RILD characterised by early (RP) and late (fibrotic) changes.

The advantage of using HU density includes its quantitative, objective and automated characterisation of RILD. There are similarities between RILD graded by traditional, physician-assessed measures and using HU density. However, the technique has important limitations. Firstly, by simply recording HU density, it is not possible to distinguish different causes for HU changes, for example tumour recurrence, infection, intravascular contrast injection or lobar collapse. Parenchymal changes are heterogenous in aetiology and morphology and to reduce them to mean HU discounts information contained in the CT scan. Simply measuring different average HU in an area of lung cannot distinguish between a small change in density at all voxels or a large increase in density across a small number of voxels. Furthermore, HU density can be confounded by a number of scan artefacts, such as the use of contrast, the presence of vessels, differing scan acquisition protocols, respiratory motion, etc. The advantage of our classification system over the ones above is that by developing a novel texture-based analysis, we are able to describe density changes with greater richness than simply using HU density.

Other groups have developed sophisticated techniques for analysing the lung parenchyma after radiotherapy [50,51] by using radiomics. While these techniques can uncover relationships between texture information in the images and the development of radiation pneumonitis and/or the radiotherapy dose, the radiomic features they use to do this do not provide a direct classification of the radiological changes, as seen in the scans by a human observer, as the tissue classes used in this work aim to do. The long-term aim of this work is to better understand the radiological manifestations of RILD, their evolution over time, and the complex relationships they have with dose, clinical outcomes, and other factors. Therefore, we have developed novel tools, such as the automated parenchymal tissue classification system presented in our accompanying paper [25], and our method for registering heavily damaged lungs [24], which will enable us to achieve a better understanding of RILD, rather than employing radiomics or modern learning-based approaches that may produce strong predictive models but do not provide any insights that can help further our understanding of RILD.

One of the potential weaknesses of our classification system is that the individual classes binned several distinct radiological entities. Class 5, for example, which represents opacity on a CT scan, could be the result of tumour, lung collapse or pleural effusion. Pleural effusions, in particular, often take up a significant proportion of the intrathoracic cavity. Being idiosyncratic and temporally unstable, they may have weakened the dosimetric correlations seen with this class. It may be helpful to introduce additional classes to represent for example, tumour, atelectasis and pleural effusion in order to further increase the utility of the classification schema. Nonetheless, we have developed a far richer classification of texture changes than that employed by using HU density alone.

The study presented in this paper is limited due to relatively low patient numbers, and therefore, did not aim to provide definitive clinical conclusions about RILD. Rather, it is a proof of principal study that aimed to demonstrate the potential of our tools to provide valuable insights into RILD, and the relationships between parenchymal tissue damage and the global and local dose and respiratory function. Another limitation of this study is that our dataset is from a trial with an isotoxic dose design, and there may be less heterogeneity between patients than in a non-trial data set, which may have masked correlations between dose and parenchymal texture. The IDEAL trial was published in 2016, and accordingly, most of the patients received 3D conformal RT. In future work, we will fully automate our methods so that they can be applied to large datasets with many patients. We will then employ these to study both large retrospective cohorts of non-trial patients, including those treated with VMAT radiotherapy [58], as well as prospective datasets that will include richer clinical data, such as patients’ reported outcome measures.

## 5. Conclusions

We have demonstrated that the recent tools we have developed for studying the radiological manifestations of RILD can provide novel insights into the temporal evolution of RILD and its relationship to global and local dose and respiratory outcomes. Our registration method for heavily damaged lungs was able to successfully register the pre-RT scan to the follow-up CT scans in 45 of 46 patients. The parenchymal tissue classes we developed demonstrated statistical correlation to both global and local dose metrics in our study, and have a distinct evolution over time. The dosimetric variables most strongly correlated with change in tissue class volume are lung V20, V30 Gy and mean lung dose. Although less strong, there is a relationship between the tissue class changes and respiratory outcomes, particularly the MRC dyspnoea score, which directly represents a patient’s functional status. We have demonstrated the potential of using our tools to analyse and understand the evolution of the radiological manifestation of RILD in greater detail than previously possible, and we hope this can ultimately be used to inform the refinement of RT treatment in order to reduce the burden of morbidity on lung cancer patients as they begin to live longer with their disease.

## Figures and Tables

**Table 1:** Patient characteristics and accompanying respiratory data.

Patient Characteristics		Number (%)	Median (Range)
Age			64 (42–83)
Gender	Male	31 (67)	
	Female	15 (33)	
AJCC Stage	IIB	3 (7)	
	IIIA	30 (65)	
	IIIB	13 (28)	
Fractionation Schedule	6 weeks	36 (78)	
	5 weeks	10 (22)	
Radiotherapy Technique	Conformal 3D	45 (98)	
	VMAT	1 (2)	
Prescribed dose (Gy)			66.75 (63–73)
PTV (Planning Tumour Volume) (cm^3^)			360.00 (139–821)
MLD (Mean Lung Dose)			14.56 (8.75–19.96)
Lung V20 Gy			22.58 (13.86–43.61)
Progression	All	20 (43)	
	Loco-regional	15 (33)	

**Table 2:** IDEAL-CRT Respiratory data available for patients included in this study.

Metric	Time Point	Available (out of 46)
FVC	Baseline	46
	3 m	43
	6 m	40
	12 m	40
	24 m	35
FEV1	Baseline	46
	3 m	43
	6 m	40
	12 m	40
	24 m	36
TLCO	Baseline	46
	3 m	40
	6 m	37
	12 m	38
	24 m	35
MRC Score	Baseline	43
	3 m	43
	6 m	43
	12 m	41
	24 m	40

**Table 3:** Lung Parenchyma classification.

Class	Description
1	Normal, healthy or emphysematous lung, without any high-density abnormality and representing most of the lung parenchymal tissue prior to radiation, as well as areas not affected during the radiotherapy.
2	Areas mostly characterised by changes similar to ground-glass opacity.
3	Areas with mixed ground-glass opacity and overlaid reticulation.
4	Mostly solid lung tissue, either aerated opaque tissue or tissue with a density just below dense opacity.
5	Homogeneous, dense lung tissue, which could represent a number of pathological entities, including tumour, pleural effusion or collapse.

## Data Availability

The data presented in this study are available on request from the corresponding author.

## Supplementary Materials

The following are available online at https://www.mdpi.com/article/10.3390/cancers14040946/s1, Figure S1: Pearson Correlation Matrix of Dosimetric metrics against Parenchymal Classes at 6 months, Figure S2: Pearson Correlation Matrix of Dosimetric metrics against Parenchymal Classes at 12 months, Figure S3: Pearson Correlation Matrix of Dosimetric metrics against Parenchymal Classes at 24 months, Figure S4: Pearson Correlation Matrix of Respiratory metrics against Parenchymal Classes at 3 months, Figure S5: Pearson Correlation Matrix of Respiratory metrics against Parenchymal Classes at 6 months, Figure S6: Pearson Correlation Matrix of Respiratory metrics against Parenchymal Classes at 12 months.
