# Supplementary material for: Quantitative Analysis of Radiation-Associated Parenchymal Lung Change
Source: Cancers (Basel). 2022 Feb 14;14(4):946. doi: 10.3390/cancers14040946 (PMC8870325; doi:10.3390/cancers14040946)
Supplement: Supplementary file 1 [file cancers-14-00946-s001.zip › cancers-1548275-supplementary.pdf]

cancers

MDPI

Supplementary Materials

Quantitative Analysis of Radiation-Associated Parenchymal Lung Change

Edward Chandy [1,2,3,*,†], Adam Szmul [1,†], Alkisti Stavropoulou [1], Joseph Jacob [1,4], Catarina Veiga [1], David Landau [2], James Wilson [5], Sarah Gulliford [5], John D. Fenwick [6], Maria A. Hawkins [5], Crispin Hiley [2,‡] and Jamie R. McClelland [1,‡]

[1] Centre for Medical Image Computing, Department of Medical Physics and Biomedical Engineering, University College London, London WC1E 6BT, UK; a.szmul@ucl.ac.uk (Adam Szmul); alkisti.stavropoulou.16@ucl.ac.uk (Alkisti Stavropoulou); j.jacob@ucl.ac.uk (J.J.); c.veiga@ucl.ac.uk (C.V.); j.mcclelland@ucl.ac.uk (J.R.M.)

[2] UCL Cancer Institute, University College London, London WC1E 6BT, UK; dblandau@gmail.com (D.L.); Crispin.Hiley@crick.ac.uk (C.H.)

[3] Sussex Cancer Centre, Royal Sussex County Hospital, Brighton BN2 5BE, UK

[4] UCL Respiratory Department, University College London Hospital, London NW1 2PG, UK

[5] Medical Physics and Biomedical Engineering, University College London, London WC1E 6BT, UK; james.wilson4@nhs.net (J.W.); s.gulliford@nhs.net (S.G.) m.hawkins@ucl.ac.uk (M.A.H.)

[6] Institute of Systems, Molecular and Integrative Biology, University of Liverpool, Liverpool L69 3GE, UK; john.fenwick@liverpool.ac.uk

Correspondence: e.chandy@nhs.net

† Joint First authors.

‡ Joint Final authors.

Supplementary Figures

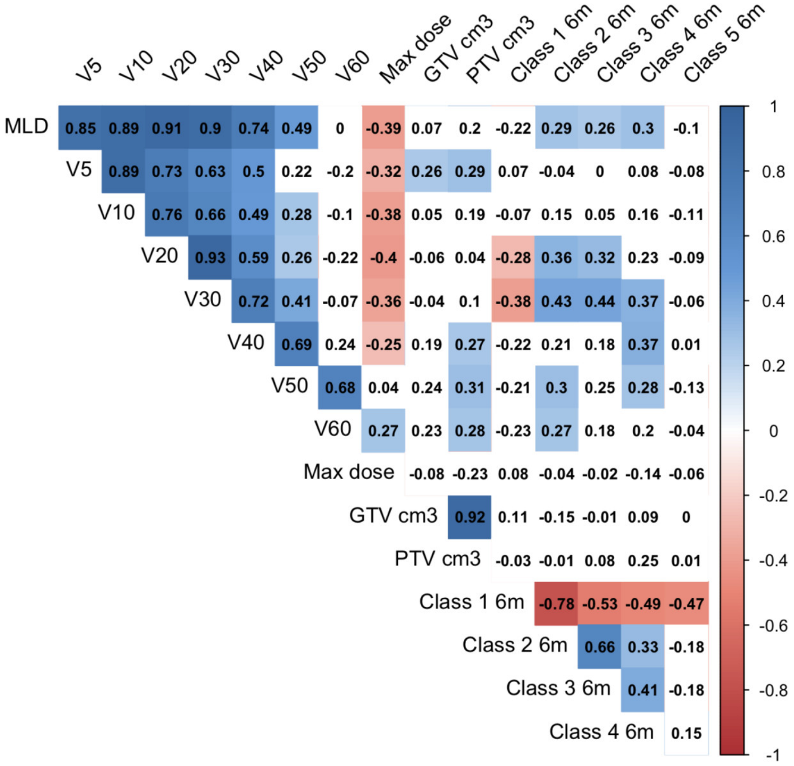

Figure S1. Pearson Correlation Matrix of Dosimetric metrics against Parenchymal Classes at 6 months.

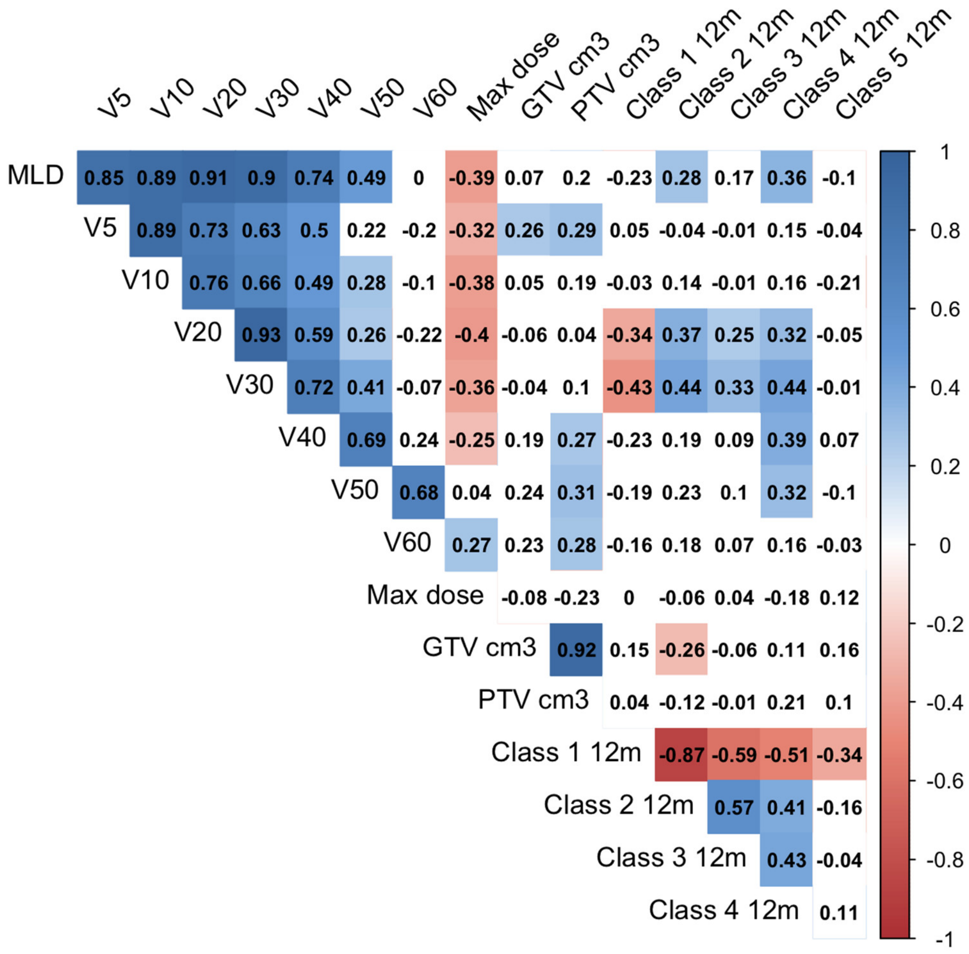

Figure S2. Pearson Correlation Matrix of Dosimetric metrics against Parenchymal Classes at 12 months.

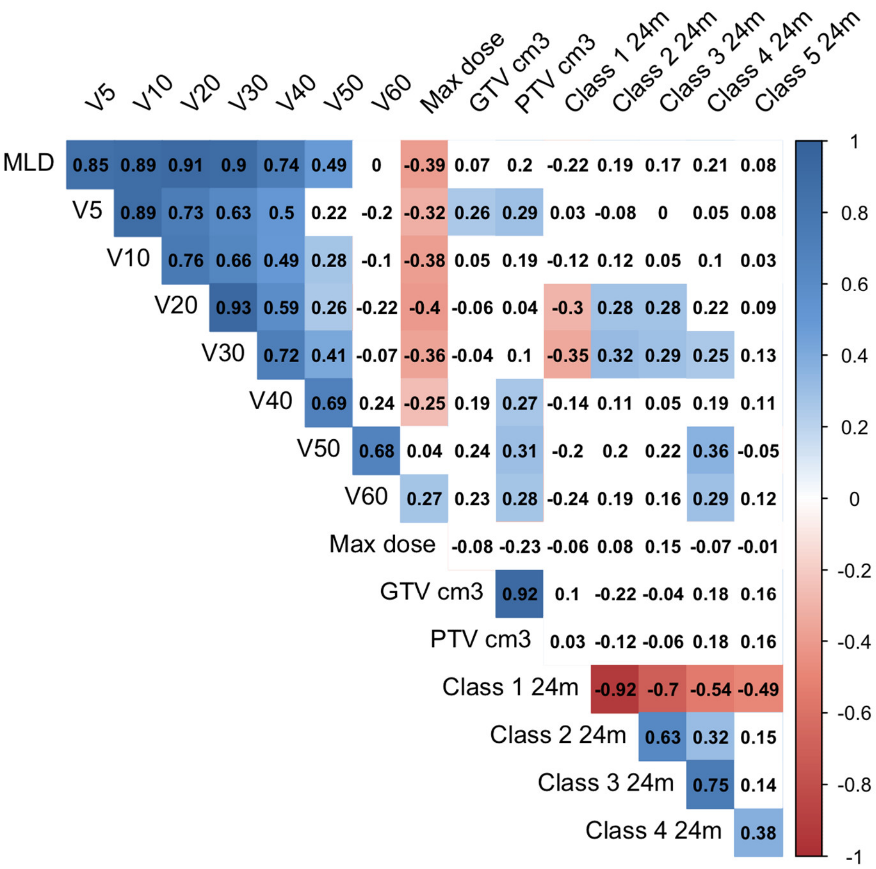

Figure S3. Pearson Correlation Matrix of Dosimetric metrics against Parenchymal Classes at 24 months.

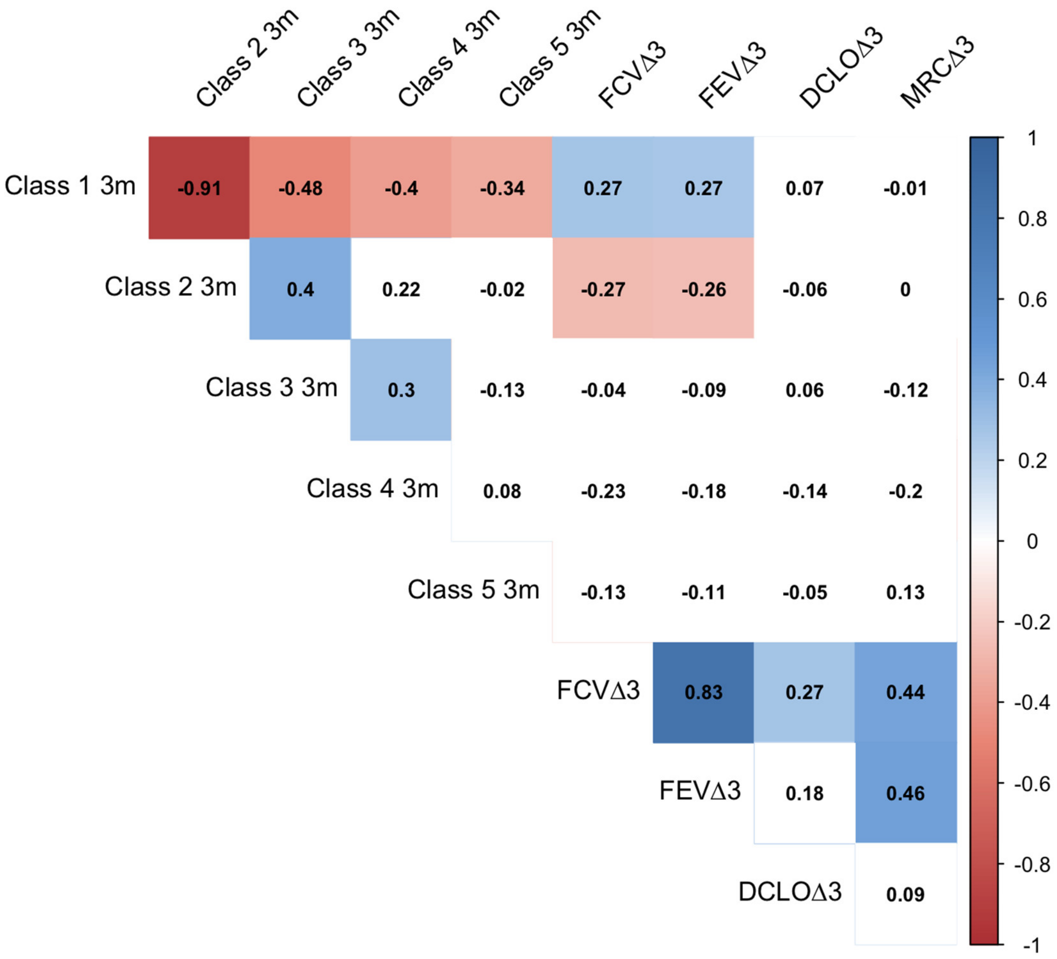

Figure S4. Pearson Correlation Matrix of Respiratory metrics against Parenchymal Classes at 3 months Figure S5 Pearson Correlation Matrix of Respiratory metrics against Parenchymal Classes at 6 months.

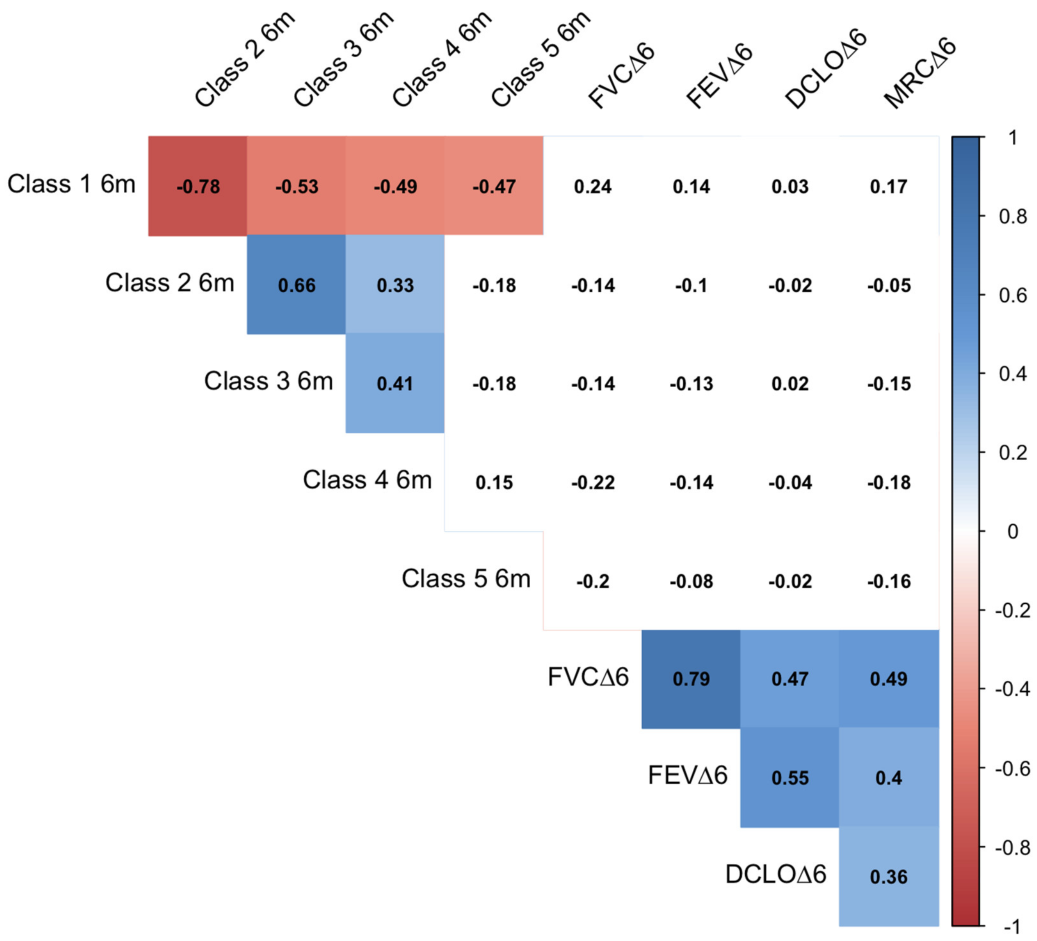

Figure S5. Pearson Correlation Matrix of Respiratory metrics against Parenchymal Classes at 6 months.

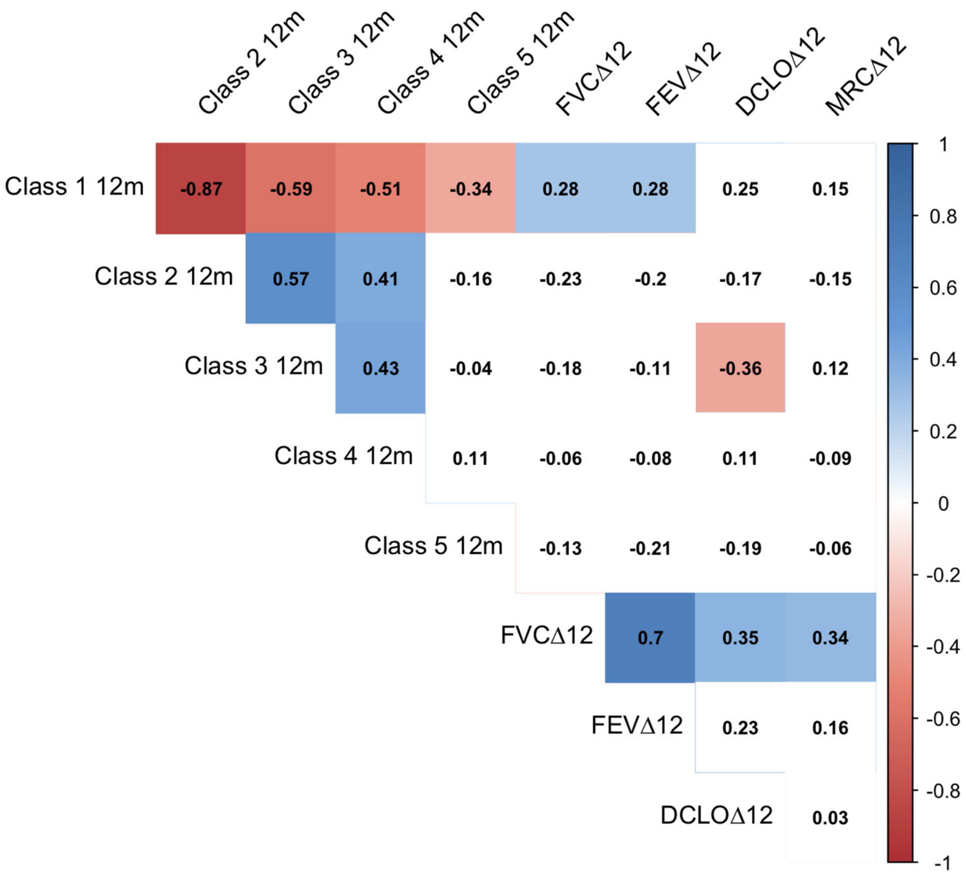

Figure S6. Pearson Correlation Matrix of Respiratory metrics against Parenchymal Classes at 12 months.
